# Effect of Reminding Patients to Complete Fecal Immunochemical Testing: A Comparative Effectiveness Study of Automated and Live Approaches

**DOI:** 10.1007/s11606-017-4184-x

**Published:** 2017-10-10

**Authors:** Gloria D. Coronado, Jennifer S. Rivelli, Morgan J. Fuoco, William M. Vollmer, Amanda F. Petrik, Erin Keast, Sara Barker, Emily Topalanchik, Ricardo Jimenez

**Affiliations:** 1 Center for Health Research, Kaiser Permanente Northwest, Portland, OR USA; 2 Sea Mar Community Health Centers, Seattle, WA USA

**Keywords:** colorectal cancer screening, fecal testing, direct-mail program, automated and live reminders, federally qualified health centers

## Abstract

**Background:**

The Community Preventive Services Task Force recommends multi-component interventions, including patient reminders, to improve uptake of colorectal cancer screening.

**Objective:**

We sought to compare the effectiveness of different forms of reminders for a direct-mail fecal immunochemical test (FIT) program.

**Design:**

Patient-randomized controlled trial.

**Participants:**

2772 adults aged 50–75, not up to date with colorectal cancer screening recommendations, with a clinic visit in the previous year at any of four participating health center clinics.

**Intervention:**

Participants were mailed an introductory letter and FIT. Those who did not complete their FIT within 3 weeks were randomized to receive (1) a reminder letter, (2) two automated phone calls, (3) two text messages, (4) a live phone call, (5) a reminder letter and a live phone call, (6) two automated phone calls and a live phone call, or (7) two text messages and a live phone call. Patients with a patient portal account were sent two email reminders, but were not randomized.

**Main Measures:**

FIT return rates for each group, 6 months following randomization.

**Key Results:**

A total of 255 (10%) participants returned their FIT within 3 weeks of the mailing. Among randomized participants (*n* = 2010), an additional 25.5% returned their FITs after reminders were delivered (estimated overall return rate = 32.7%). In intention-to-treat analysis, compared to the group allocated to receive a reminder letter, return rates were higher for the group assigned to receive the live phone call (OR = 1.51 [1.03–2.21]) and lower for the group assigned to receive text messages (OR = 0.66 [0.43–0.99]). Reminder effectiveness differed by language preference.

**Conclusions:**

Our data suggest that FIT reminders that included a live call were more effective than reminders that relied solely on written communication (a text message or letter).

Trial Registration: ClinicalTrials.gov/ctc2/show/NCT01742065.

## INTRODUCTION

Colorectal cancer (CRC) is the second leading cause of cancer death in the United States.^1^ In 2017, an estimated 135,000 individuals will be diagnosed with CRC, and 50,000 will die from the disease.^1^ While screening for CRC has been shown unequivocally to reduce incidence and mortality,^2^ adherence to screening remains low. In 2015, only 63% of adults aged 50 and older reported being up to date with CRC screening recommendations.^1^ Screening rates are particularly low among Hispanics (50%), uninsured individuals (25%), and immigrants who have been in the United States for fewer than 10 years (34%). In community health centers providing care to underserved patients, an average of only 38% of eligible adults were found to be up to date in 2015.^3^ Direct-mail fecal testing programs have been shown to improve CRC screening rates in multiple health care settings, including community health centers,^2^^,^ 4 ^–^ 9 with improvements ranging from a 22% to 45% absolute increase.^2^^,^ 4 ^–^ 9 Most of these programs delivered follow-up reminders to adults who were mailed fecal test kits, with the reminders generally delivered through a letter or telephone call (either automated or live) or, more recently, through text messages and occasionally patient navigation (staff trained to provide assistance with overcoming screening barriers).^2^^,^ 4 ^,^ 8 ^–^ 11

Despite the widespread use of follow-up reminders for direct-mail fecal testing programs, few studies have evaluated the impact that reminders have on fecal test return rates. To address this important gap in the literature, we compare the effectiveness of reminders for a direct-mail fecal testing program. We partnered with Sea Mar Community Health Centers, the largest community health center in the state of Washington, to answer the question: How well do individual and multiple reminders work for patients who are mailed a fecal immunochemical test (FIT)? A secondary question was: Can we infer a difference in the effectiveness based on patients’ preferred language (English or Spanish)? We also report on the percentage of eligible patients who received the reminders (reach). The results of this study will inform future efforts to optimize the return rate for direct-mail fecal testing programs.

## METHODS

### Study Setting

We partnered with Sea Mar Community Health Centers to compare the effectiveness of multiple strategies to remind patients who were mailed a FIT to mail it back. Sea Mar is a health center in western Washington that serves more than 130,000 patients in 32 medical clinics. We selected four Sea Mar primary care clinics for our pilot. In 2015, Sea Mar served more than 29,000 patients aged 50 to 75. Sea Mar’s CRC overall screening rate in 2015 was 40%.

### Reminders for a Direct-Mail Program

**Figure 1:**
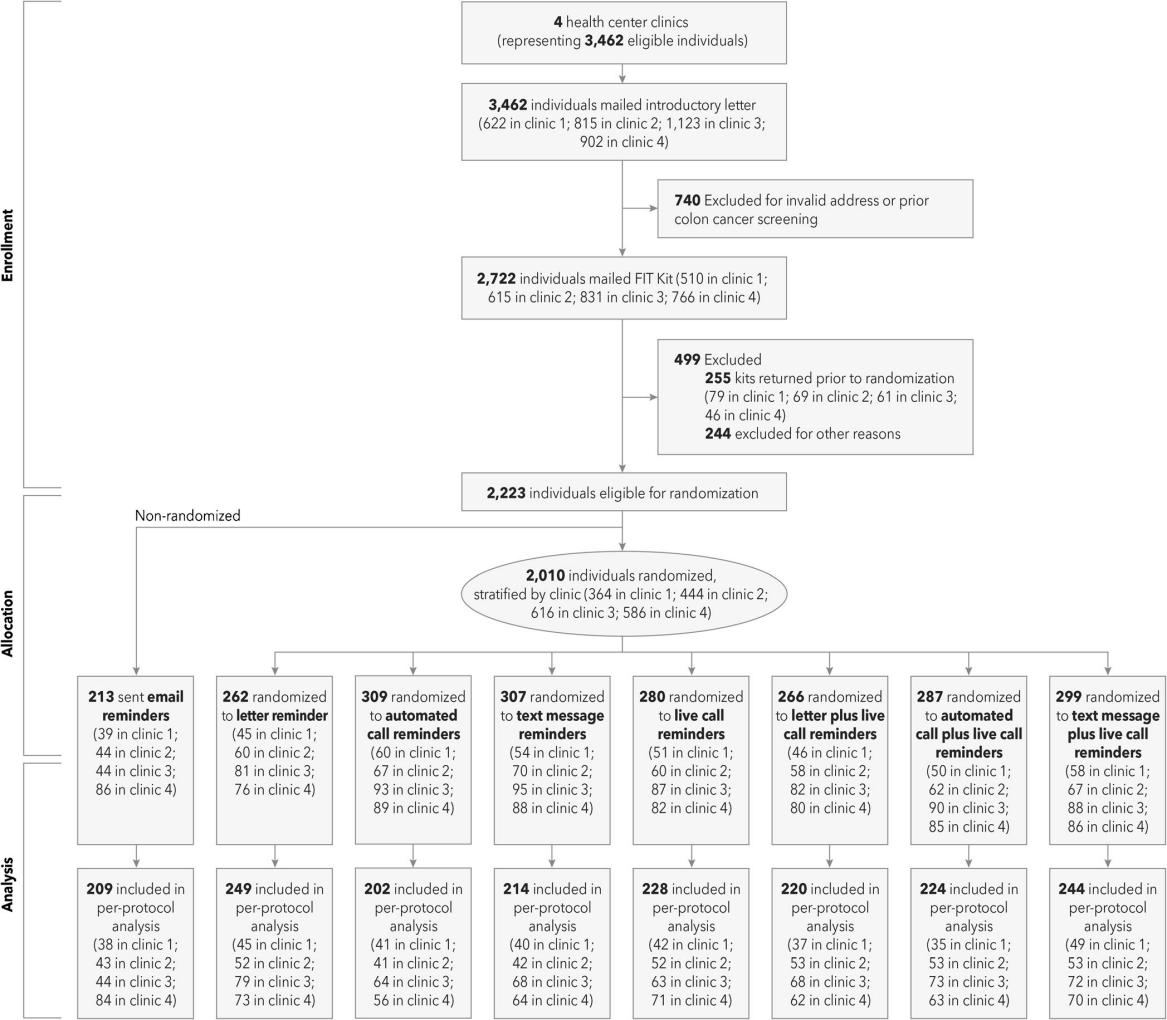
CONSORT diagram of Sea Mar.

This pilot study compared the effectiveness of four single-mode reminders (a reminder letter, automated phone calls, live phone calls, and text messages) and three multi-modal reminders (a reminder letter and live call, automated and live calls, and text messages and live call). Our pilot also included a separate parallel non-randomized arm to determine the feasibility of delivering reminders using an electronic patient portal (Fig. 1). Patients with active patient portal accounts were not randomized and were sent reminder messages through the portal. All procedures and intervention materials were reviewed and approved by the institutional review board of Kaiser Permanente Northwest, and the study was reviewed and approved by Sea Mar’s research committee. Informed consent was waived.

We mailed the introductory letter and FIT kit packets to all eligible adults using templates developed for the Strategies and Opportunities to Stop Colorectal Cancer (STOP CRC) program.^12^ The introductory letter explained the importance of CRC screening and informed recipients they would be mailed a FIT. The letters were developed in English and translated into Spanish and Russian. FIT kit packets contained a FIT kit, pictorial wordless^13^ and written instructions (in English, Spanish) on how to complete the kit, and a postage-paid envelope for returning the kit to the health center’s centralized laboratory. All screening was conducted using the Polymedco OC FIT-Chek (Polymedco, Inc., Cortlandt Manor, NY), a single-specimen FIT that requires no dietary restrictions.

Research and clinic staff developed reminders using materials adapted from the National Colorectal Cancer Roundtable.^14^ All written and automated reminders (e.g., reminder letter, automated phone calls, text messages, patient portal messages) were developed in English and translated into Spanish and Russian by certified bilingual project staff. The script for the live call was developed in English and translated into Spanish by bilingual project staff (none of the outreach workers spoke Russian).

### Study Procedures

Centralized clinic outreach staff used electronic health record codes developed and refined in STOP CRC to identify adults overdue for CRC screening who had attended a clinic visit within the calendar year.^15^ A total of 3462 adults met the criteria and were mailed an introductory letter. Clinic staff identified anyone whose letter was returned by the post office as undeliverable, and reviewed the medical records to identify anyone who was up to date with CRC screening (not captured using health record codes), and removed 740 names. The remaining 2722 adults were mailed a FIT kit (2 weeks later). An additional 244 patients were excluded after the mailing because they reported prior CRC screening, declined participation or no longer received care at Sea Mar, or had an invalid address (not previously identified).

Patients who returned their FITs within 3 weeks (*n* = 255) and who had patient portal accounts (*n* = 213) were not randomized. Those with patient portal accounts were sent two email reminders to return their FITs. The remaining 2010 were stratified by clinic and randomized (in a 1:1 ratio) into one of the seven intervention groups via a random number algorithm generated by the project analyst. Clinic outreach staff assigned eligible participants to the intervention in sequential order. Reminders were delivered on a set schedule (Table 1). Live calls were delivered by bilingual patient advocates fluent in English and Spanish, and interpreter services were available for patients who spoke other languages. Live calls were made between the hours of 9:30 a.m. and 5:00 p.m. All automated calls, text messages, and patient portal messages were delivered by the health center’s vendor, ClientTell (Valdosta, GA). For automated phone calls, patients were invited to press “0” to be transferred immediately to speak to a Sea Mar staff person. To lessen the burden on clinic staff, the four clinics launched the intervention at 7-week intervals from November 2015 to May 2016.

**Table 1:** Schedule of Delivery and Reach by Reminder Mode

Randomization status	No. of reminders	Week delivered*	Reach
			% (denominator)
Randomized			
Overall			78.7 (2010)
Reminder letter	1	0	95.0 (262)
Automated phone call ^†^	2	0, 3	65.4 (309)
Text message ^‡^	2	0, 3	69.7 (307)
Live phone call ^§^	2	0, 3	81.4 (280)
Reminder letter /	1	0	82.7 (266)
Live phone call	2	3, 5	
Automated phone call /	2	0, 1	78.0 (287)
Live phone call	2	3, 5	
Text message /	2	0, 1	81.6 (299)
Live phone call	2	3, 5	
Non-randomized			
Patient portal	2	0, 3	98.1 (213)

*Week following randomization

^†^ Patients were considered reached by an automated phone call if a message was left or the call was answered

^‡^ Patients were considered reached by a text message if their phone number in the medical record was a cell phone. No further information was available about whether a patient received a text message

^§^ Six patients had missing outcomes for live-call reminders. Of these, five had evidence of a completed FIT. These patients were kept in the per-protocol analysis to avoid bias

### Analysis

We describe the demographic characteristics of all randomized patients across each intervention arm, the percentage of FIT kits returned after the FIT mailing and prior to randomization, and the percentage of FIT kits returned within 6 months of randomization. Our primary intention-to-treat analysis compared FIT return rates across treatment arms using a 6-degrees-of-freedom Wald test. We performed the same test according to language subgroup. If the Wald test was significant (*p* < 0.05), we used logistic regression, adjusting for clinic, to compare the effects in each treatment arm, including the patient portal arm, to the reminder letter reference group. We did not adjust the paired analyses for multiple comparisons, because our findings were considered exploratory.

For the per-protocol analysis sample, we excluded patients whose reminder letters were returned by the post office as undeliverable and those who had disconnected or non-working phone numbers, among other reasons. Sea Mar’s vendor relayed whether an automated message was left on a recipient’s voice mail or whether the call was answered in person. Clinic outreach staff entered telephone numbers into an online search engine to determine whether the number was assigned to a landline or cell phone. Patients receiving text messages who had landlines were excluded from the per-protocol sample (*n* = 93; 30%). The vendor provided no additional information as to whether patients actually received the text messages.

## RESULTS

A total of 2010 patients were randomized to one of the seven reminder arms (Fig. 1). Randomized patients were mostly aged 50–64 years, and men and women were represented in similar proportions (Table 2). Hispanics represented about one-quarter of the sample, and nearly 20% of the sample preferred speaking Spanish. Over three-quarters of the sample were insured, and about one-half had family income less than $20,000.

**Table 2:** Demographic Characteristics of Eligible Adults in Intention-to-Treat Analysis Sample*

		Randomized							Non-randomized
	Overall (*N* = 2010)	Reminder letter (*n* = 262)	Automated call (*n* = 309)	Text message (*n* = 307)	Live phone call (*n* = 280)	Reminder letter / Live phone call (*n* = 266)	Automated phone call/ Live phone call (*n* = 287)	Text message / Live phone call (*n* = 299)	Patient portal message (*n* = 213)
	% (no.)	%	%	%	%	%	%	%	%
Age (years)									
50–64	82.1 (1651)	80.9	82.2	80.5	85.0	81.6	83.6	81.3	84.0
65–74	17.9 (359)	19.1	17.8	19.5	15.0	18.4	16.4	18.7	16.0
Gender									
Female	52.4 (1054)	52.3	53.1	51.8	52.9	50.4	52.6	53.8	52.1
Male	47.6 (956)	47.7	46.9	48.2	47.1	49.6	47.4	46.2	47.9
Ethnicity^†^									
Hispanic	24.2 (487)	24.8	22.7	25.1	27.5	22.2	20.6	26.8	7.5
Non-Hispanic	75.0 (1507)	74.4	77.3	73.6	71.4	76.3	79.4	72.2	92.0
Language									
English	73.0 (1467)	71.0	74.4	69.7	71.1	75.6	75.3	73.9	91.5
Spanish	19.1 (384)	21.0	16.8	21.2	22.9	15.0	15.7	21.1	3.3
Other	7.9 (159)	8.0	8.7	9.1	6.1	9.4	9.1	5.0	5.2
Insurance status									
Medicaid/Medicare	74.8 (1504)	74.8	74.8	73.0	72.5	75.9	74.2	78.6	80.8
Uninsured	14.3 (288)	14.1	14.9	14.3	14.6	12.8	16.0	13.4	5.6
Commercial	10.1 (202)	9.9	10.0	12.1	11.1	10.9	8.7	7.7	12.2
Family income									
<$20,000	49.8 (1001)	50.0	47.2	51.1	52.1	48.1	49.8	50.2	43.2
$20,000+	13.4 (270)	11.1	13.3	15.6	15.4	11.3	16.0	11.0	14.1
Unknown	36.8 (739)	38.9	39.5	33.2	32.5	40.6	34.1	38.8	42.7

*Sample excludes patients who returned fecal test before reminders were delivered

^†^Ethnicity unknown for 16 participants

Overall, 512 randomized adults returned their FIT kits, for a return rate of 25.5%. When we include those who returned their FIT kits within the 3 weeks prior to randomization and those in the patient portal group, the estimated overall return rate was 32.7%. In intention-to-treat analysis, patients allocated to live phone call reminders had 50% greater odds of completing their FIT kits than patients allocated to reminder letters (Table 3). In contrast, patients who received text-message reminders had 34% lower odds of returning their FITs than patients who received mailed letters. We found no significant differences in FIT completion rates for other reminder protocols compared to a simple reminder letter. A total of 21% of individuals in the patient portal group returned their FIT kits.

**Table 3:** FIT Test Completion by Reminder Mode, Using Intention-to-Treat Analysis Sample*

	FIT completion					
	Overall		Language preference			
Reminder mode	% (denominator) OR (95% CI)		English % (denominator) OR (95% CI)		Spanish % (denominator)	Other % (denominator)
Randomized						
Total	25.5 (2010)		22.4 (1467)		34.1 (384)	33.3 (159)
Reminder letter	23.7 (262)	Ref	18.8 (186)	Ref	32.7 (55)	42.9 (21)
Automated phone call	23.3 (309)	0.99 (0.67, 1.46)	22.6 (230)	1.28 (0.79, 2.07)	25 (52)	25.9 (27)
Text message	16.9 (307)	0.66 (0.43, 0.99)	13.6 (214)	0.67 (0.39, 1.15)	23.1 (65)	28.6 (28)
Live phone call	31.8 (280)	1.51 (1.03, 2.22)	29.6 (199)	1.83 (1.14, 2.96)	37.5 (64)	35.3 (17)
Reminder letter / Live phone call	27.4 (266)	1.22 (0.83, 1.81)	23.9 (201)	1.35 (0.83, 2.20)	35 (40)	44 (25)
Automated phone call / Live phone call	28.9 (287)	1.32 (0.90, 1.93)	23.6 (216)	1.33 (0.82, 2.16)	48.9 (45)	38.5 (26)
Text message / Live phone call	27.1 (299)	1.21 (0.83, 1.78)	24.4 (221)	1.42 (0.88, 2.29)	39.7 (63)	13.3 (15)
Non-randomized						
Patient portal message	20.7 (213)	0.87 (0.56, 1.35)	19.0 (195)	1.01 (0.61, 1.69)	28.6 (7)	45.5 (11)

*Sample excludes patients who returned fecal test before reminders were delivered; OR based on logistic regression analysis adjusting for clinic, and reported only if overall 6-*df* Wald test for comparing randomized groups was significant at *p* < 0.05

In per-protocol analysis (Table 4), overall FIT return was highest in the groups that received either the live phone calls (OR, 1.73; [95% CI, 1.16–2.58]) or the combination of automated and live calls (OR, 1.74; [95% CI, 1.17–2.60]). In per-protocol analyses stratified by language preference, English-preferring adults had higher odds of FIT return if they were allocated to a live call (OR, 2.17; [95% CI, 1.31–3.59]), a combination of a reminder letter and live call (OR, 1.78; [95% CI, 1.07–2.97]), an automated and live call (OR, 1.73; [95% CI, 1.04–2.88]), or text message and live call (OR, 1.90; [95% CI, 1.15–3.14]). Among Spanish-preferring adults, higher odds of FIT return were associated with the combination of automated and live phone calls (OR, 3.45; [95% CI, 1.42–8.39]).

**Table 4:** FIT Test Completion by Reminder Mode, Using Per-Protocol Analysis Sample*

	FIT completion					
	Overall		Language preference			
Reminder mode	% (denominator) OR (95% CI)		English % (denominator) OR (95% CI)		Spanish % (denominator) OR (95% CI)	
Randomized						
Total	28.7 (1581)		25.3 (1149)		37.7 (308)	
Reminder letter	23.7 (249)	Ref	18.4 (174)	Ref	32.7 (55)	Ref
Automated phone call	25.2 (202)	1.10 (0.71, 1.69)	24 (154)	1.42 (0.83, 2.43)	30.3 (33)	0.92 (0.36, 2.34)
Text message	16.4 (214)	0.63 (0.40, 1.00)	14.2 (148)	0.73 (0.40, 1.32)	18.6 (43)	0.49 (0.19, 1.27)
Live phone call	34.6 (228)	1.73 (1.16, 2.58)	32.7 (165)	2.17 (1.31, 3.59)	38.5 (52)	1.31 (0.59, 2.91)
Reminder letter / Live phone call	32.7 (220)	1.57 (1.04, 2.35)	28.8 (163)	1.78 (1.07, 2.97)	37.8 (37)	1.25 (0.52, 3.00)
Automated phone call / Live phone call	35.3 (224)	1.74 (1.17, 2.60)	28.3 (166)	1.73 (1.04, 2.88)	61.1 (36)	3.45 (1.42, 8.39)
Text message / Live phone call	32.4 (244)	1.56 (1.05, 2.33)	29.6 (179)	1.90 (1.15, 3.14)	46.2 (52)	1.81 (0.82, 3.99)
Non-randomized						
Patient portal message	21.1 (209)	0.88 (0.57, 1.37)	19.4 (191)	1.06 (0.63, 1.80)	28.6 (7)	1.00 (0.17, 5.81)

*Sample excludes patients who returned fecal test before reminders were delivered or who were not reached by a given reminder mode; OR based on logistic regression analysis adjusting for clinic, and reported only if overall 6-*df* Wald test for comparing randomized groups was significant at *p* < 0.05

In total, 79% of randomized adults were reached; reach was highest for the patient portal (98%) and reminder-letter groups (95%) and lowest for the text-message (70%) and automated-call (65%) groups (Table 1). Among the 202 adults reached via automated phone call, 62% received a voice message and 38% were reached in person. Among the 228 reached via the live call, 50% were reached in person.

## DISCUSSION

Previous research has acknowledged the success of direct-mail fecal testing programs and the limitations of point-of-care methods for delivery of fecal testing.^5^ We assessed the effectiveness of automated reminders and combined automated and live reminders for a direct-mail FIT program in a large, diverse community health center in western Washington. While many direct-mail fecal testing programs have delivered patient reminders, ours is the first study to rigorously test the effectiveness of these reminders in a community health center population, and among patients with differing language preferences.

We observed important variations in return rates based on reminder mode used, and both the overall return rate and reminder mode effectiveness differed according to patients’ language preferences. In general, modes that included a live call performed better than modes that relied solely on written communication (a text message or letter). Despite our effort to translate written and automated reminders for the three most common languages spoken by patients in the health center (English, Spanish, and Russian), low levels of health literacy are thought to be common among patients served by community health centers. For patients who preferred speaking Spanish, the combination of the automated and live phone calls produced the highest return rates. A live call may help build trust (*confianza*), an important value and motivator for care-seeking among Latinos.

Overall, patients who received live reminder phone calls were more likely to complete a FIT than those who were mailed reminder letters; this finding is consistent with some previous evaluations and inconsistent with others. Among Latino and Vietnamese patients in a large public hospital, Walsh and colleagues reported a significant boost in self-reported fecal testing rates among patients who received a mailed fecal test and telephone counseling compared to those who received only a mailed fecal test (absolute percentages in each group: 25% vs. 15%; *p* < 0.01).^16^ In contrast, Levy and colleagues tested the effectiveness of a direct-mail FIT program targeting 373 consented participants in rural primary practices in Iowa, with and without a structured live reminder phone call delivered by project staff, and found no additional boost in FIT returns (absolute percentages in each group: 45% vs. 49%; *p* = 0.50).^17^ Our findings suggest that a live call is superior to a mailed reminder in improving FIT returns.

Contrary to expectations, we observed lower odds of FIT return among adults assigned to receive text-message reminders, and the effect was unchanged in per-protocol analysis where landline phone numbers were removed from the denominator. Baker and colleagues and Gupta and colleagues used text-message reminders in direct-mail FIT programs; however, both combined this approach with concurrently delivered automated phone calls, making it unclear how to attribute the effect.^2^^,^ 5 However, in sub-analyses limited to those who did and did not receive a text-message reminder, Baker and colleagues reported no difference in return rates (44% vs. 44%), suggesting minimal influence from this approach.^2^ Our findings show that text messages, by themselves, performed more poorly than reminder letters.

We observed that randomized patients who preferred Spanish returned their FITs at a higher rate than patients who preferred English (FIT return rates: 34% v. 22%, *p* < 0.001; data not shown). This finding may reflect the relatively low likelihood that Latino patients will discuss CRC screening with their doctors during in-clinic visits.^14^ Our research team previously reported a higher FIT return rate for Spanish speakers than English speakers in a direct-mail FIT program delivered to patients in a community health center (FIT return rate: 46% vs. 28%, respectively).^11^ Similarly, Gupta and colleagues reported higher direct-mail FIT return rates by Hispanics than non-Hispanic whites (FIT return rate: 48% vs. 34%, respectively), although the differences were not directly tested.^5^ In contrast, Baker and colleagues reported no differences in the magnitude of the effect of direct-mail and reminder interventions based on race/ethnicity or preferred language.^2^

Program reach for follow-up reminders is an important consideration, especially in community health centers, where patients’ contact information may become quickly outdated. Among the limited number of studies conducted in community health centers, Jean-Jacques and colleagues reported that 40% of patients were reached after three live phone attempts, and that 23% of available phone numbers were either incorrect or disconnected.^8^ Similarly, Singal and colleagues and Baker and colleagues separately reported that only 48% and 37% of intended recipients, respectively, were reached with up to three attempts to deliver a live-call reminder.^2^^,^ 9 We observed that 81% of intended recipients were reached using up to two attempts to deliver a live call. Previous studies have reported that automated phone calls were successfully delivered to 76–89% of intended recipients,^2^^,^ 4 ^,^ 18 with over half (51–54%) of the calls answered by a voice message system.^2^^,^ 4 We observed lower reach for automated phone calls in our study (65%). Text messages were successfully delivered to 51% of recipients in a study by Baker and colleagues and to 78% of recipients in a study by Goldman and colleagues.^2^^,^ 4 We observed a 70% reach for text messages. Our findings underscore the importance of maintaining up-to-date telephone contact information for the delivery of both automated and live telephone reminders.

The effectiveness of reminders must be weighed against the costs of delivering those reminders and patient experience with receiving reminders, among other factors. Automated phone calls and text messages are the least costly options to implement, yet live reminders may allow staff to address or triage other patient health care needs. Future research might assess the views of clinic leadership and patients regarding the value of FIT reminders.

### Strengths and Limitations

Our study had a number of strengths, including its large and diverse sample, randomized design, and the near-complete capture of fecal testing events and demographic characteristics in the electronic health record. In addition, we used a waiver of informed consent to avoid selection bias. The delivery of live reminders by bilingual (English and Spanish) clinic staff also meant that the quality of intervention delivery did not differ measurably between patients whose preferred language was English or Spanish. Moreover, our delineation of phone numbers as cell or landline numbers allowed us to uniquely assess return rates among patients in the text-message group who had cell phones.

Notwithstanding these strengths, however, the study did have several limitations. Relatively few patients in our sample had active patient portal accounts, and the non-randomized allocation to this group meant that we were unable to more rigorously assess the effectiveness of this modality compared to other reminder modes. Some aspects of our available data limited our ability to ascertain whether a patient received the reminder(s). Additionally, it is possible that letters sent to invalid addresses may not always have been returned and that automated phone messages could have been delivered to unintended recipients. Also, even though we determined whether phone numbers were cell or landline numbers, we could not be certain that cell phones enabled text-message functions. We have no way of knowing the magnitude of this misclassification.

## CONCLUSION

Our FIT kit reminder study involved adults receiving care at a community health center in the Pacific Northwest. Our findings show that there are variations in FIT return rates based on reminder mode used and that both the overall return rate and effectiveness of reminder mode differs according to patient language preference. Our data appear to suggest that reminders that included a live call performed better than reminders that relied on written communication (a text or letter).
